# Endothelial cells enhance adipose mesenchymal stromal cell‐mediated matrix contraction via ALK receptors and reduced follistatin: Potential role of endothelial cells in skin fibrosis

**DOI:** 10.1002/jcp.26494

**Published:** 2018-05-09

**Authors:** Hanneke N. Monsuur, Lenie J. van den Broek, Pieter Koolwijk, Frank B. Niessen, Susan Gibbs

**Affiliations:** ^1^ Department of Dermatology, VU University Medical Center Amsterdam Movement Sciences Amsterdam The Netherlands; ^2^ Department of Physiology, Amsterdam Cardiovascular Sciences VU University Medical Center Amsterdam The Netherlands; ^3^ Department of Plastic, Reconstructive and Hand Surgery VU University Medical Center Amsterdam The Netherlands; ^4^ Department of Oral Cell Biology, Academic Center for Dentistry Amsterdam (ACTA), University of Amsterdam and VU University Amsterdam Amsterdam Movement Sciences Amsterdam The Netherlands

**Keywords:** endothelial cells, fibrosis, skin, scar

## Abstract

Abnormal cutaneous wound healing can lead to formation of fibrotic hypertrophic scars. Although several clinical risk factors have been described, the cross‐talk between different cell types resulting in hypertrophic scar formation is still poorly understood. The aim of this in vitro study was to investigate whether endothelial cells (EC) may play a role in skin fibrosis, for example, hypertrophic scar formation after full‐thickness skin trauma. Using a collagen/elastin matrix, we developed an in vitro fibrosis model to study the interaction between EC and dermal fibroblasts or adipose tissue‐derived mesenchymal stromal cells (ASC). Tissue equivalents containing dermal fibroblasts and EC displayed a normal phenotype. In contrast, tissue equivalents containing ASC and EC displayed a fibrotic phenotype indicated by contraction of the matrix, higher gene expression of ACTA2, COL1A, COL3A, and less secretion of follistatin. The contraction was in part mediated via the TGF‐β pathway, as both inhibition of the ALK4/5/7 receptors and the addition of recombinant follistatin resulted in decreased matrix contraction (75 ± 11% and 24 ± 8%, respectively). In conclusion, our study shows that EC may play a critical role in fibrotic events, as seen in hypertrophic scars, by stimulating ASC‐mediated matrix contraction via regulation of fibrosis‐related proteins.

**Abbreviations**
α‐SMA: α‐smooth muscle actin
ASC: adipose tissue‐derived mesenchymal stromal cell
BMP: bone morphogenic protein
CTGF: connective tissue growth factor
EC: endothelial cells
Fib: dermal fibroblast
GDF: growth differentiation factor
MSC: mesenchymal stromal cells
TGF‐β: transforming growth factor‐β
TIMP‐1: tissue metalloproteinase‐1

## 1 INTRODUCTION

Abnormal wound healing of the skin can lead to the formation of fibrotic hypertrophic scars which show, for example, redness, itch, pain, and joint contracture. Hypertrophic scars remain within the boundaries of the original wound and are usually formed after extreme skin trauma, for example, full‐thickness burns, but can also occur after standard surgical procedures. For example, 1 year after full‐thickness burn injury up to 72% of burn patients have hypertrophic scars and 1 year after standard surgery 35% of patients have hypertrophic scars (Bloemen et al., 2009; Lawrence, Mason, Schomer, & Klein, 2012; Mahdavian Delavary, van der Veer, Ferreira, & Niessen, 2012; Niessen, Spauwen, Robinson, Fidler, & Kon, 1998; van der Veer et al., 2011). Since wounds that form hypertrophic scars are generally full‐thickness wounds it is thought that cells from the adipose tissue may contribute to their development (Matsumura et al., 2001; van den Bogaerdt et al., 2009). Although several risk factors have been described such as size, depth, and delayed wound closure, the cross‐talk between different cell types resulting in hypertrophic scar formation are still poorly understood (Gangemi et al., 2008).

Normal cutaneous wound healing consists of multiple overlapping phases (Reinke & Sorg, 2012). Immediately after wounding, a fibrin clot is formed which acts as a provisional matrix. This permits an influx of neutrophils and monocytes into the wound bed thus initiating an inflammatory cascade. During the proliferation phase, re‐epithelialization takes place and granulation tissue is formed. Granulation tissue is formed by an accumulation of fibroblasts, capillaries (endothelial cells), immune cells, and collagen bundles. An important part of normal wound healing involves the replacement of the granulation tissue with extracellular matrix and apoptosis of excessive numbers of fibroblasts and endothelial cells (EC) (Johnson & DiPietro, 2013). Apoptosis of EC ensures that overabundant small blood vessels regress and enables maturation of newly formed networks. Due to the complexity of wound healing, many steps along the way are prone to aberrations and have been described to lead to the formation of hypertrophic scars. For example, delayed re‐epithelialization, prolonged inflammation, excessive neovascularization, imbalance of matrix metalloproteinases and their inhibitors, and prolonged presence of myofibroblasts causing excessive extracellular matrix deposition are all related to an increased chance of hypertrophic scar formation (DiPietro, 2016; Mustoe & Gurjala, 2011; Zhu, Ding, & Tredget, 2016). Also, differences in the organization of the collagen bundles in granulation tissue, where mesenchymal stromal cells (MSC) and EC play an important role, can discriminate between normotrophic scars and hypertrophic scars (Linares, 1996). Previously we described a hypertrophic scar model in which adipose tissue‐derived mesenchymal stromal cells (ASC), when incorporated into a skin equivalent, caused contraction and a hypertrophic phenotype (Boink et al., 2016; van den Broek, Niessen, Scheper, & Gibbs, 2012). Several studies indicate that changes in vascularization or endothelial dysfunction may play a role in hypertrophic scar formation or regression, respectively (Amadeu et al., 2003; van der Veer et al., 2011; Wang, Song, & Liu, 2017 Xi‐Qiao, Ying‐Kai, Chun, & Shu‐Liang, 2009). Also in other organs, for example, in liver and lung, EC have been implicated in formation of fibrotic tissue (Elpek, 2015; Farkas, Gauldie, Voelkel, & Kolb, 2011). Taken together this suggests that both ASC and EC may be involved in the onset of hypertrophic scar formation.

Transforming growth factor‐β1 (TGF‐β1) secreted by, for example, platelets, macrophages, keratinocytes, and fibroblasts is associated with fibrosis and scarring (Barrientos, Stojadinovic, Golinko, Brem, & Tomic‐Canic, 2008; Lichtman, Otero‐Vinas, & Falanga, 2016). Increased TGF‐β stimulates fibrosis by binding to the ALK5 receptor (TGFβR1) and TGFβR2 and subsequently upregulating type 1 collagen and tissue inhibitor of metalloproteinase‐1 (TIMP‐1) gene expression and downregulating matrix metalloproteinase‐1 gene expression in fibroblasts leading to enhanced matrix deposition and impaired degradation of extracellular matrix components (Baum & Arpey, 2005; Ghahary, Shen, Scott, & Tredget, 1995; Verrecchia & Mauviel, 2007). Furthermore, other TGF‐β family members have been described as fundamental regulators of inflammation and fibrosis in several organs, such as activin A and B, growth differentiation factor (GDF) 8 and bone morphogenetic proteins (BMP) 2 and 6 (Cantini et al., 2008; Hedger & de Kretser, 2013; Kaiser et al., 1998; Stelnicki et al., 1998; Werner & Alzheimer, 2006). Activin A, which binds to ALK4 (ACTRIB) and ACTRII or IIB, is thought to exert its effect in fibrosis by upregulation of fibrotic factors TGF‐β1, connective tissue growth factor (CTGF), TIMP‐1, plasminogen activator inhibitor‐1, endothelin and type 1 collagen (Hedger & de Kretser, 2013). Interestingly, activin A expression was detected in inflammatory cells during the early phases of burn wound healing, while it was predominantly seen in fibroblasts during later phases of burn wound healing (McLean et al., 2008). Anti‐fibrotic follistatin is primarily known to bind and neutralize activin A with high affinity, but is also able to bind with lower affinity to activin B, GDFs, and BMPs (Amthor et al., 2004; Glister, Kemp, & Knight, 2004; Harrington et al., 2006; Otsuka, Moore, Iemura, Ueno, & Shimasaki, 2001; Schneyer et al., 2008). These results suggest that activin A, next to TGF‐β, plays a key role in fibrosis starting already in the inflammatory and granulation tissue phase of cutaneous wound healing.

Although many studies implicate a role for either ASC or EC in hypertrophic scar formation, the interaction between both cell types and the cross‐talk mechanism by which they might stimulate dermal matrix contraction and fibrosis has not been studied. The aim of this study was to investigate the crosstalk between MSC (fibroblast or ASC) and EC in fibrosis in vitro by seeding the cells into a 3D collagen/elastin dermal matrix (MatriDerm®). MSC and EC derived from the dermis were compared with those derived from the adipose tissue in order to determine the influence of wound depth on in vitro fibrosis.

## 2 MATERIALS AND METHODS

### 2.1 Human tissue

Human adult skin with underlying adipose tissue was obtained anonymously from healthy individuals undergoing abdominal dermolipectomy. Tissue collection procedures were performed in compliance with the “Code for Proper Secondary Use of Human Tissue” as formulated by the Dutch Federation of Medical Scientific Societies (http://www.federa.org) and following procedures approved by the institutional review board of the VU University medical center.

### 2.2 Cell culture

Adipose tissue was carefully dissected from the skin. The remaining skin was then treated with dispase to remove the epidermis from the dermis. The adipose stromal vascular cell fraction and dermal stromal vascular cell fraction were then isolated using collagenase type II/dispase II from adipose tissue or dermis as previously described (Kroeze et al., 2009).

#### 2.2.1 Mesenchymal stromal cells

ASC and fibroblasts, passage 3, were obtained with 99.9% purity (CD90+, CD31−) and cultured in DMEM (Lonza, Verviers, Belgium), 1% UltroSerG (UG) (BioSepra SA, Cergy‐Saint‐Christophe, France) and 1% penicillin/streptomycin (P/S) (Invitrogen, Gibco, Paisley, UK) (Kroeze et al., 2009).

#### 2.2.2 Endothelial cells

EC were purified from the adipose stromal vascular cell fraction (A‐EC) and from the dermal stromal vascular cell fraction (D‐EC) using a MidiMACS separator with microbeads against CD31 (Monsuur et al., 2016). A >99% pure population (CD31+/CD90−) was obtained at passage 3. EC were cultured on 1% gelatin (Sigma‐Aldrich, St. Louis, MO) coated flasks in EC medium: M199 medium (Lonza), 1% P/S, 2 mM L‐glutamin (Invitrogen,Carlsbad, CA), 10% heat‐inactivated New Born Calf Serum (Invitrogen), 10% heat‐inactivated Human Serum (Invitrogen), 5 U/ml heparin (Pharmacy VUmc, Amsterdam, The Netherlands) and 3.75 μg/ml endothelial cell growth factor (crude extract from bovine brain) (Physiology department VUmc, Amsterdam, The Netherlands).

The cells were stored in liquid nitrogen until required. For experiments ASC and fibroblasts between passage 2 and 3 were used and EC between passage 5 and 7. In all experiments donor‐matched cells were used.

### 2.3 Culture of tissue equivalents

#### 2.3.1 Tissue equivalents

Transwells (0.4 μm, Corning, NY) containing collagen/elastin matrix (MatriDerm®; Dr. Suwelack Skin & Health Care, Billerbeck, Germany) were used to create tissue equivalents as previously described (van den Broek et al., 2012). This dermal matrix was chosen since it is currently used underneath split‐skin autografts for treating full‐thickness burns (van Zuijlen et al., 2015). ASC, fibroblasts, adipose‐EC or dermal‐EC (4.10^5^) were seeded into the collagen/elastin matrix (22 × 22 mm) either alone or in combinations of ASC or fibroblasts with EC (4.10^5^ + 4.10^5^). The tissue equivalents were cultured submerged for 3 weeks in EC medium with addition of 50 μg/ml ascorbic acid and 5 ng/ml epidermal growth factor (Sigma‐Aldrich). Culture medium was replaced twice a week. Twenty four hours before harvesting, culture medium was replaced by EC medium without heparin, ECGF and epidermal growth factor for ELISA. The tissue equivalents were harvested for immunohistochemical analysis and mRNA expression.

#### 2.3.2 Addition of TGF‐β1 pathway inhibitors to culture medium

Tissue equivalents were generated as described above with culture medium being supplemented with ALK4/5/7 inhibitor (2 or 10 μM SB431542; Sigma‐Aldrich), or recombinant follistatin (0.5 ng/ml follistatin; R&D Systems, Abingdon, UK). Corresponding controls were supplemented with 0.04% DMSO or 0.5% PBS containing 1% BSA.

### 2.4 Measurement of matrix contraction

Photographs of the tissue equivalents were taken at the start and end of the experiments with a Nikon Coolpix 5400 digital camera (Japan). The surface areas of the equivalents were measured by NIS‐Elements AR 2.10 software.

### 2.5 Immunohistochemical analysis

Immunohistochemical analysis was performed on paraffin embedded sections (5 µm) for CD31 (endothelial cells, clone JC70A; 1:40, DAKO, Glostrup, Denmark), Vimentin (mesenchymal cells, clone V9; 1:200, DAKO) and α‐SMA (myofibroblasts, clone 1A4; 1:200, DAKO).

### 2.6 Secretion of granulation tissue formation factors, cytokines, and chemokines

ELISAs were performed using commercially available ELISA antibodies. All reagents were used in accordance to the manufacturer's specifications. TGF‐β1, activin A, follistatin (all R&D Systems). ELISA data are expressed in ng/ml.

### 2.7 mRNA expression

Upon harvesting the tissue equivalents, a small piece (40–100 mm^2^) was snap frozen and stored at −80°C. The tissue equivalents were homogenized using gentleMACS™ M Tubes in combination with the gentleMACS Dissociators according to manufacturer's instructions (Miltenyi Biotec, Bergisch Gladbach, Germany). Further homogenization was performed using the QiaShredder kit and RNA isolation was performed using the RNeasy Mini kit with on‐column DNAse digestion (Qiagen, Hilden, Germany) according to the manufacturer's instructions. Reverse transcription of RNA and the real‐time PCR reactions were performed essentially as previously described (van der Meijden et al., 2014) using SYBRGreen iQ™ SYBR® Green Supermix (Bio‐Rad Laboratories, Hercules, CA) and qPCR primer pairs for ACTA2 (α‐smooth muscle actin; HP205437, OriGene Technologies, Rockville), COL1A1 (Collagen 1; HP200074, OriGene), COL3A1 (Collagen 3; HP200076, OriGene), TGFB1 (TGF‐β HP200609, OriGene) or housekeeping gene HPRT1 (HP200179, OriGene). Gene expression (2^−ΔCt^) was normalized for the expression of housekeeping gene HPRT1.

### 2.8 Statistical analysis

Statistical analyses were performed using *t*‐tests or one‐way ANOVA for measurements of contraction or ELISA results. For the PCR data a one‐way ANOVA was used followed by a Kruskal Wallis test. All data were obtained from three to seven independent experiments using different donors and duplicate wells. The EC and MSC in each experiment were donor‐matched. Differences were considered significant when *p < 0.05, **p < 0.01, ***p < 0.001. Results are shown as mean ± SEM.

## 3 RESULTS

### 3.1 Co‐culture of EC with ASC results in pronounced matrix contraction

**Figure 1:**
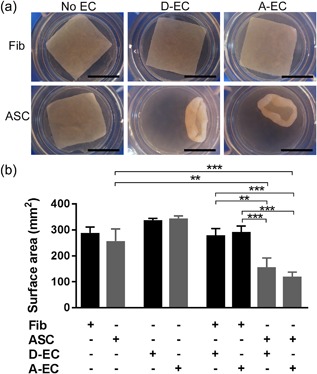
EC derived from either the dermal or adipose tissue induce contraction of tissue equivalents containing ASC, but have no effect on tissue equivalents containing fibroblasts. (a) Macroscopic pictures of tissue equivalents (bars = 1 cm). (b) Surface area of tissue equivalent with Fibroblasts (Fib), ASC, D‐EC, A‐EC or combinations between Fib, ASC, and EC. Statistical analysis was performed using repeated measures one‐way ANOVA, analysis with tissue equivalents containing only EC was not included. **p < 0.01, ***p < 0.001. Data are shown for three donors (EC alone) or four donors (all other conditions) as mean ± SEM. A‐EC, adipose‐endothelial cells; ASC, adipose‐tissue derived mesenchymal stromal cells; D‐EC, dermal‐endothelial cells; Fib, fibroblasts

**Figure 2:**
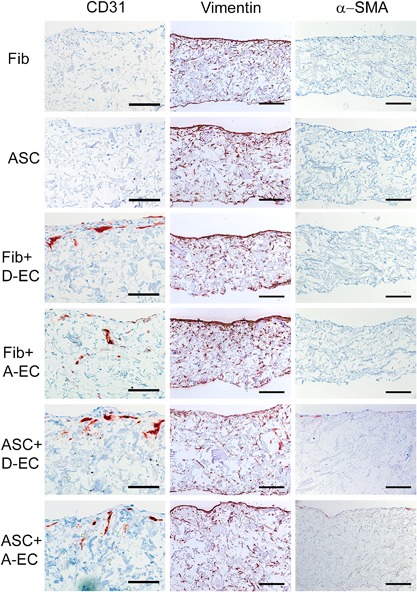
Microscopic assessment of tissue equivalents. Immunohistochemical staining of CD31, Vimentin and α‐SMA (bars = 100 μm)

Since contraction is a striking feature of fibrosis and hypertrophic scars, it was first determined whether different combinations of EC and MSC derived from dermis and adipose tissue could influence the surface areas of a 3D collagen/elastin matrix. These tissue equivalents containing either dermal fibroblasts or ASC in mono‐culture resulted in only moderate contraction (15 ± 6% and 26 ± 13% contraction, respectively) (Figures 1a and 1b). Tissue equivalents containing only dermal‐EC (D‐EC) or adipose‐EC (A‐EC) did not contract. However, tissue equivalents containing ASC in co‐culture with either dermal‐EC or adipose‐EC showed very strong contraction (55 ± 9% and 66 ± 4%, respectively). This enhanced contraction was not observed when dermal fibroblasts were co‐cultured with either dermal‐EC or adipose‐EC. Non‐contracted tissue equivalents remained flattened whereas the contracted tissue equivalents were curved at the edges (Figure 1) and were thicker (Figure 2). Immunohistochemical analysis of the endothelial marker CD31 showed that EC were generally located in the upper half of the tissue equivalent (Figure 2). EC were clustered and often resembled micro‐vessel‐like structures. In contrast, MSC (vimentin staining) were localized throughout the entire matrix with no obvious clustering. No α‐smooth muscle actin (α‐SMA) positive cells were seen in the tissue equivalents containing only fibroblasts or ASC indicating the absence of myofibroblasts. Tissue equivalents consisting of fibroblasts co‐cultured with dermal‐EC or adipose‐EC showed only sporadic α‐SMA staining. Surprisingly, the most contracted tissue equivalents, those containing ASC with adipose‐EC or ASC with dermal‐EC, showed only low numbers of α‐SMA positive cells.

Taken together these results show that EC derived from either the dermis or adipose tissue induced contraction of tissue equivalents containing ASC, but had no effect on tissue equivalents containing fibroblasts.

### 3.2 Higher mRNA expression of fibrosis‐related genes during co‐culture of adipose‐EC with ASC

**Figure 3:**
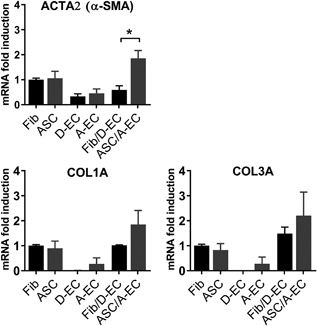
Expression of fibrosis‐related genes in tissue equivalents. Relative mRNA expression of ACTA2, COL1A, and COL3A by tissue equivalents compared to fibroblasts (Fib). Statistical analysis was performed using a one‐way ANOVA followed by a Kruskal‐Wallis test. *p < 0.05. Data are shown for two donors (D‐EC) or four donors (Fib/EC, ASC/EC, A‐EC) or five donors (Fib, ASC) as mean ± SEM. A‐EC, adipose‐endothelial cells; ASC, adipose‐tissue derived mesenchymal stromal cells; D‐EC, dermal‐endothelial cells; Fib, fibroblasts

Since relatively little α‐SMA expressing cells were observed in tissue equivalents, even in the contractile ones where EC were co‐cultured with ASC, it was next determined whether the gene expression of ACTA2 (encoding α‐SMA), COL1A1 (encoding collagen 1) and COL3A1 (encoding collagen 3) was altered since these genes have been described to play key roles in excessive matrix formation and hypertrophic scar formation (Sidgwick & Bayat, 2012; van der Veer et al., 2009; Xi‐Qiao et al., 2009) (Figure 3). ACTA2 was expressed more by fibroblasts and ASC compared to dermal‐ and adipose‐EC. Moreover, ACTA2 expression was significantly upregulated by tissue equivalents containing co‐cultured adipose‐EC and ASC compared to co‐cultures containing dermal‐EC and fibroblasts. A similar trend was seen for COL1A and COL3A. Overall, the tissue equivalent containing co‐cultured adipose‐EC and ASC showed higher COL1A, COL3A, and ACTA2 than the other tissue equivalents.

### 3.3 EC stimulate ASC to contract the matrix via the ALK4, ALK5, and ALK7 receptors and reduction of follistatin secretion

**Figure 4:**
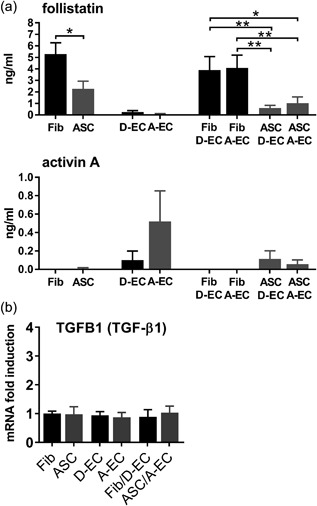
Secretion and expression of pro‐ and anti‐fibrotic factors. (a) Secretion of anti‐fibrotic follistatin and pro‐fibrotic activin A by tissue equivalents. Fibroblasts (Fib), ASC, D‐EC, and A‐EC each contain 4 × 10^5^ cells. Co‐cultures of Fib/ASC and D‐EC/A‐EC contain 8 × 10^5^ (4 × 10^5^ + 4 × 10^5^) cells. (b) mRNA expression of TGFB1 by tissue equivalents. mRNA data are shown relative to Fib. Statistical analysis was performed using *t*‐tests or repeated measures one‐way ANOVA. *p < 0.05, **p < 0.01. Secretion data are shown for three donors (EC alone) or four donors (all other conditions) as mean ± SEM. mRNA data are shown for two donors (D‐EC) or four donors (Fib/EC, ASC/EC, A‐EC) or five donors (Fib, ASC) as mean ± SEM. A‐EC, adipose‐endothelial cells; ASC, adipose‐tissue derived mesenchymal stromal cells; D‐EC, dermal‐endothelial cells; Fib, fibroblasts

To study the underlying molecular pathways involved in the observed contraction of the tissue equivalents containing ASC and EC we next measured secretion of follistatin, activin A, and mRNA expression of TGF‐β1 (Figure 4). Anti‐fibrotic follistatin binds and neutralizes pro‐fibrotic activin A and with less affinity also other TGF‐β family members. The tissue equivalents containing ASC with or without EC (independent of EC origin) secreted less follistatin than the tissue equivalents containing fibroblasts with or without EC (Figure 4a), indicating a more pro‐fibrotic environment in the tissue equivalents containing ASC. The effect is most pronounced when ASC are co‐cultured with EC (independent of EC origin). Activin A secretion was detected in equivalents containing D‐EC, A‐EC, or ASC‐EC (Figure 4a). In all tissue equivalents, TGF‐β1 secretion was below the detection limit of our ELISA (30 pg/ml; data not shown) therefore we measured the mRNA expression, which was similar in all conditions (Figure 4b).

**Figure 5:**
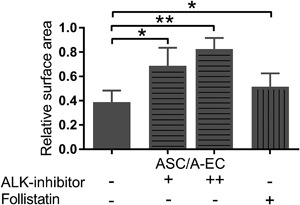
EC stimulate ASC to contract the matrix via follistatin and the ALK4, ALK5, and ALK7 receptors. Surface area when cultured in the absence or presence of 2 μM (+) or 10 μM (++) ALK‐inhibitor SB431542 or 0.5 ng/ml (+) rh‐follistatin, relative to surface area at time 0. Statistical analysis was performed using *t*‐tests or repeated measures one‐way ANOVA. *p < 0.05, **p < 0.01. Data are shown for four donors as mean ± SEM. A‐EC, adipose‐endothelial cells; ASC, adipose‐tissue derived mesenchymal stromal cells; D‐EC, dermal‐endothelial cells; Fib, fibroblasts

In order to further investigate the role of follistatin in our in vitro fibrosis model a broad inhibition of the TGF‐β pathway was obtained by blocking the ALK4, ALK5, and ALK7 receptors with the compound SB431542 (Inman et al., 2002). In tissue equivalents containing co‐cultured ASC and EC, inhibition of the ALK4/5/7 receptors resulted in dose dependent inhibition of matrix contraction leading to almost complete inhibition (75 ± 11%) (Figure 5). Since follistatin was more expressed in non‐contracting tissue equivalents containing fibroblasts (+/− EC) than in contracting tissue equivalents containing ASC‐EC we next determined whether the addition of follistatin to ASC‐EC tissue equivalents could prevent matrix contraction (Figure 5). Indeed, contraction was significantly decreased by 24 ± 8% when follistatin was added to the culture medium.

Taken together, these results indicate that EC, independent of their dermal or adipose origin, stimulate ASC to contract the matrix via the ALK4, ALK5, and ALK7 receptors of the TGF‐β pathway and reduction of follistatin secretion.

## 4 DISCUSSION

The aim of this in vitro study was to investigate whether EC may play a role in skin fibrosis, for example, hypertrophic scar formation after deep skin trauma, where healing arises from the exposed adipose tissue. Using a collagen/elastin matrix routinely used under split‐thickness autografts in burn patients (van Zuijlen et al., 2015), we developed an in vitro fibrosis model to study the specific interaction between EC and dermal fibroblasts or ASC, without interference of other cell types present in the skin. EC, regardless of their origin (dermal or adipose tissue), stimulated ASC to contract the matrix. The contraction was in part mediated via the TGF‐β pathway, as both inhibition of the ALK4/5/7 receptors by SB431542 and the addition of recombinant follistatin resulted in decreased contraction of the matrix. Altogether our results strengthen the hypothesis that EC contribute to hypertrophic scar formation.

**Figure 6:**
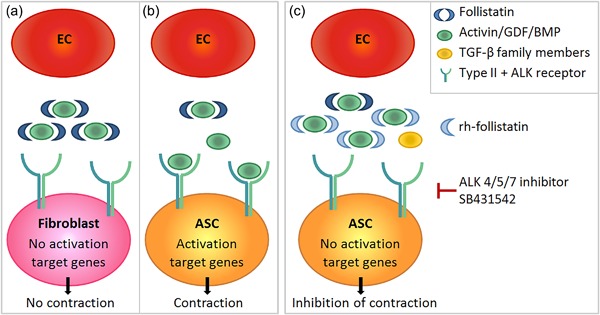
Mechanism of EC enhanced ASC‐mediated matrix contraction. (a) Follistatin secreted by fibroblasts sequesters specific members of the TGF‐β family, such as activin A, preventing activin A from activating the ALK4 receptor and the downstream fibrogenic target genes, thereby preventing dermal contraction. (b) ASC secrete less follistatin than fibroblasts. Therefore follistatins pro‐fibrotic targets are insufficiently sequestered and residual activin/GDF/BMP can activate the ALK4 receptor and the downstream target genes, leading to dermal matrix contraction and fibrosis. (c) By blocking the pathways of the ALK 4/5/7 receptors with SB431542, the proteins of the TGF‐β family are prevented from binding to these receptors, leading to a large inhibition of matrix contraction. Addition of recombinant follistatin prevents the binding of activin A to the ALK4 receptor, leading to decreased matrix contraction, but to a lesser extent than SB431542. A‐EC, adipose‐endothelial cells; ASC, adipose‐tissue derived mesenchymal stromal cells; D‐EC, dermal‐endothelial cells; Fib, fibroblasts; TGF‐β, transforming growth factor‐β

In Figure 6 our proposed scheme of the mechanism by which EC enhance ASC‐mediated matrix contraction is shown with follistatin with anti‐fibrotic properties and activin A with pro‐fibrotic properties (Hedger & de Kretser, 2013). Here we show that fibroblasts, isolated from the dermis, secrete more follistatin than ASC isolated from the adipose tissue. Follistatin sequesters members of the TGF‐β family, such as activin A, thus preventing activin A from activating the ALK4 receptor and its downstream fibrogenic target genes. This would explain, in part, why little contraction and fibrosis occurs in superficial wounds (normotrophic scar) which only extend into the dermis. In contrast, we show that ASC, by secreting less follistatin than fibroblasts, are unable to sufficiently sequester members of the TGF‐β family, such as activin A thus permitting the activation of the ALK4 receptor and its downstream target genes on ASC, leading to contraction and fibrosis. By blocking the pathways of ALK 4/5/7 receptors on ASC the proteins of the TGF‐β family are prevented from binding to these receptors, leading to almost total inhibition of matrix contraction. Addition of recombinant follistatin, could only prevent the binding of activin A to the ALK4 receptor on ASC, thus leaving the receptors still available for other TGF‐β family members, with the result that only partial inhibition of contraction was observed.

The contraction of the tissue equivalents which we observed when ASC were co‐cultured with EC in the matrix, while no contraction was observed when ASC were cultured alone was already evident within the first week of the 3 week culture period. In line with these findings, we previously showed that a reconstructed epidermis grown on the same ASC‐populated matrix also resulted in significant contraction whereas the matrix containing only ASC only marginally contracted within the 5 week culture period (Boink et al., 2016; van den Broek et al., 2012). This contraction, in the presence of the reconstructed epidermis coincided with upregulation of α‐SMA directly under the epidermis indicating that the ASC had become myofibroblasts. In our current study we showed that although not many α‐SMA positive cells were present in the tissue sections, we did find an upregulation of ACTA2 (α‐SMA) at mRNA level. This suggests that either a longer time in co‐culture with EC or additional factors from, for example, keratinocytes are required for ASC to fully differentiate into myofibroblasts. It has also been shown that MSC are able to contract tissue in an α‐SMA independent manner, via microfilaments (Dawes et al., 2008; Shinde, Humeres, & Frangogiannis, 2017).

TGF‐β, activin A and follistatin have been implicated in contraction and fibrosis (Leask & Abraham, 2004; Ohga, Matsuse, Teramoto, & Ouchi, 2000; Werner & Alzheimer, 2006). By blocking the ALK4/5/7 receptors we observed an almost total inhibition of matrix contraction of tissue equivalents containing both ASC and EC indicating an important role for the TGF‐β family. TGF‐β1 mRNA was expressed similar by all conditions while TGF‐β1 secretion was not detected in the culture supernatants most probably due to direct uptake by neighboring cells. In line with our results, which show a role for EC in enhancing ASC‐mediated matrix contraction via the TGF‐β pathway, it has been suggested that apoptotic EC can stimulate skin fibrosis via TGF‐β regulated CTGF secretion (DiPietro, 2016; Laplante et al., 2010). We also investigated the role of follistatin and activin A in our model. Activin A, though in small amounts, was secreted by EC independent of their dermal or adipose origin. EC being the source of activin A secretion could explain why we only observed contraction in the co‐cultures of ASC with EC as opposed to ASC alone. Others found activin A secretion by ASC when grown in co‐culture with EC, leading to ASC differentiation into smooth muscle cells (Merfeld‐Clauss et al., 2014). Moreover, the anti‐fibrotic follistatin was secreted more by tissue equivalents containing fibroblasts and EC than tissue equivalents containing ASC and EC. Our finding that recombinant follistatin reduced contraction of tissue equivalents containing ASC and EC is in line with others who observed a positive effect of follistatin on cutaneous wound healing in mice, where the addition of follistatin inhibited dermal scar formation (Bamberger et al., 2005). Although follistatin binds activin A with the strongest affinity it is possible that follistatin also exerts its anti‐fibrotic effect via sequestering of other proteins, such as activin B, GDF 8, 9, and 11 or BMP 4, 6, 7, and 15 (Amthor et al., 2004; Glister et al., 2004; Harrington et al., 2006; Otsuka et al., 2001; Schneyer et al., 2008). However, the role in skin fibrosis of these proteins has not been investigated in detail.

In conclusion, our study shows that EC may play a critical role in fibrotic events, as seen in hypertrophic scars, by stimulating ASC‐mediated matrix contraction via reduction of follistatin. Further research into the regulatory function of follistatin in fibrosis could lead to new targets to improve or prevent skin fibrosis such as hypertrophic scars arising from deep wounds where adipose tissue is exposed.

## CONFLICTS OF INTEREST

We have the following interests: Susan Gibbs is co‐founder of A‐Skin BV which is a VU university medical center startup company (SME).
